# Chronic Kidney Disease as a Risk Factor for Heart Failure With Preserved Ejection Fraction: A Focus on Microcirculatory Factors and Therapeutic Targets

**DOI:** 10.3389/fphys.2019.01108

**Published:** 2019-09-04

**Authors:** Jens van de Wouw, Michelle Broekhuizen, Oana Sorop, Jaap A. Joles, Marianne C. Verhaar, Dirk J. Duncker, A. H. Jan Danser, Daphne Merkus

**Affiliations:** ^1^Division of Experimental Cardiology, Department of Cardiology, Erasmus MC University Medical Center, Rotterdam, Netherlands; ^2^Department of Internal Medicine, Erasmus MC University Medical Center, Rotterdam, Netherlands; ^3^Division of Neonatology, Department of Pediatrics, Erasmus MC University Medical Center, Rotterdam, Netherlands; ^4^Department of Nephrology and Hypertension, University Medical Center Utrecht, Utrecht, Netherlands

**Keywords:** chronic kidney disease, heart failure, HFpEF, vasculature, microcirculation, therapy

## Abstract

Heart failure (HF) and chronic kidney disease (CKD) co-exist, and it is estimated that about 50% of HF patients suffer from CKD. Although studies have been performed on the association between CKD and HF with reduced ejection fraction (HFrEF), less is known about the link between CKD and heart failure with preserved ejection fraction (HFpEF). Approximately, 50% of all patients with HF suffer from HFpEF, and this percentage is projected to rise in the coming years. Therapies for HFrEF are long established and considered quite successful. In contrast, clinical trials for treatment of HFpEF have all shown negative or disputable results. This is likely due to the multifactorial character and the lack of pathophysiological knowledge of HFpEF. The typical co-existence of HFpEF and CKD is partially due to common underlying comorbidities, such as hypertension, dyslipidemia and diabetes. Macrovascular changes accompanying CKD, such as hypertension and arterial stiffening, have been described to contribute to HFpEF development. Furthermore, several renal factors have a direct impact on the heart and/or coronary microvasculature and may underlie the association between CKD and HFpEF. These factors include: (1) activation of the renin-angiotensin-aldosterone system, (2) anemia, (3) hypercalcemia, hyperphosphatemia and increased levels of FGF-23, and (4) uremic toxins. This review critically discusses the above factors, focusing on their potential contribution to coronary dysfunction, left ventricular stiffening, and delayed left ventricular relaxation. We further summarize the directions of novel treatment options for HFpEF based on the contribution of these renal drivers.

## Introduction

Heart failure with preserved ejection fraction (HFpEF) is characterized by impaired relaxation of the left ventricle (LV) during diastole and accounts for over 50% of all patients with heart failure (HF) (Redfield et al., 2003; Yancy et al., 2006). Both the proportion of HFpEF-patients and morbidity, mortality, and healthcare costs associated with this disease are rising (Bhatia et al., 2006; Liao et al., 2006; Owan et al., 2006; Steinberg et al., 2012). Multiple processes including cardiomyocyte hypertrophy, interstitial fibrosis, impaired calcium handling, and increased passive cardiomyocyte stiffness contribute to the left ventricular stiffening characteristic for HFpEF (Borlaug, 2014; Gladden et al., 2014; Sharma and Kass, 2014). Although ejection fraction is still normal, systolic dysfunction is present in HFpEF, as measured by tissue Doppler or strain imaging (Borlaug, 2014; Tadic et al., 2017). In large population studies, the majority of the HFpEF patients are women (Masoudi et al., 2003). Whereas men have more coronary artery disease indicative of macrovascular disease, women typically present with obesity, left ventricular hypertrophy, diastolic dysfunction and more often have microvascular angina (Gori et al., 2014a; Crea et al., 2017).

The current paradigm for HFpEF proposes that commonly present comorbidities such as diabetes mellitus (DM), obesity, and hypertension lead to a systemic pro-inflammatory state. This pro-inflammatory state causes coronary microvascular dysfunction, evidenced by an imbalance between nitric oxide (NO) and reactive oxygen species (ROS) leading to stiffening of the LV (Paulus and Tschope, 2013; Gladden et al., 2014). Excessive ROS-production in the endothelium of the coronary microvasculature lowers NO bioavailability through scavenging of NO. Loss of NO reduces soluble guanylate cyclase (sGC) activity in the cardiomyocytes, thereby lowering cGMP levels and decreasing PKG activity. The latter results in hypophosphorylation of titin and induces cardiomyocyte hypertrophy (Paulus and Tschope, 2013; Franssen et al., 2016). Given the proposed central role for disruption of the NO pathway in pathogenesis of HFpEF, it is rather surprising that all large clinical trials, which targeted the NO-cGMP-PKG pathway failed to date. Organic and inorganic nitrates are therapeutic agents that can be metabolized to NO systemically and thus act as NO-donors. However, the NEAT-HFPEF trial showed that isosorbide mononitrate, a long working organic nitrate, tended to reduce physical activity and did not improve quality of life and exercise capacity (Redfield et al., 2015). Inhaled nebulized inorganic nitrate, also did not improve exercise capacity, as recently shown in the INDIE-HFpEF trial (Borlaug et al., 2018). The phase 2b SOCRATES-PRESERVED trial showed no reduction of NT-pro-BNP or left atrial dimensions at 12 weeks after treatment with the sGC stimulator Vericiguat. However, Vericiguat was well tolerated and increased quality of life, warranting further research (Pieske et al., 2017). Inhibition of the cGMP-degrading enzyme phosphodiesterase 5 with Sildenafil did not improve clinical status rank score or exercise capacity (Redfield et al., 2013), and failed to improve vascular and cardiac function (Borlaug et al., 2015). Therefore, new therapeutic targets need to be identified that can interfere with the development and progression of HFpEF.

It is important to note that the impact of microvascular dysfunction on cardiac structure and function is not limited to dysfunction of the NO-cGMP-PKG pathway. Indeed, upregulation of VCAM-1 and E-selectin on the coronary microvascular endothelium induces transendothelial leucocyte migration and activation, increased transforming growth factor β (TGF-β) levels, thereby promoting pro-fibrotic pathways and differentiation of fibroblast to myofibroblasts (Westermann et al., 2011; Paulus and Tschope, 2013) and increasing interstitial fibrosis (van Heerebeek et al., 2012; Sharma and Kass, 2014). Secretion of autocrine and paracrine factors, such as apelin, TGF-β, and endothelin-1, by dysfunctional coronary microvascular endothelial cells can also directly induce left ventricular hypertrophy (Kamo et al., 2015). Finally, capillary rarefaction and inadequate angiogenesis could contribute to a decreased oxygen supply and subsequent left ventricular myocardial stiffening (Gladden et al., 2014).

The so-called cardio-renal syndrome describes the co-existence of HF and chronic kidney disease (CKD). Approximately 50% of the patients with HFpEF also suffer from CKD (Ter Maaten et al., 2016). Although this co-existence is partially due to shared risk factors, such as hypertension, DM and obesity, it has also been proposed that HF directly impacts kidney function, and vice versa, CKD worsens cardiac function (Brouwers et al., 2013). Interdependence of the heart and kidneys, similarities between their microvascular networks, and the coexistence of CKD and HF further imply a role for microvascular dysfunction in development and progression of both diseases (Ter Maaten et al., 2016).

**Figure 1:**
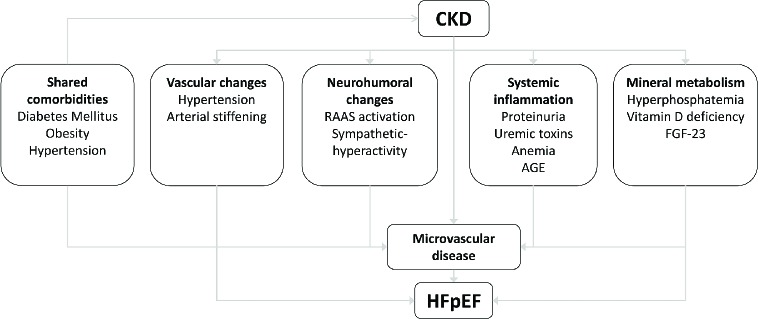
Schematic overview of the risk factors that can contribute to the development of heart failure with preserved ejection fraction (HFpEF) in patients with chronic kidney disease (CKD).

Given the co-incidence of HFpEF and CKD, the present review aims to provide a mechanistic link between CKD and HFpEF, by describing potential pathways through which CKD can induce or aggravate coronary microvascular dysfunction and thereby contribute to the development and progression of left ventricular hypertrophy and diastolic dysfunction. These include mechanical effects, neurohumoral activation, systemic inflammation, anemia and changes in mineral metabolism as induced by CKD (Figure 1). As some of these CKD-induced effects may induce HFpEF and contribute to cardiovascular disease in general, they may provide targets to intervene with the development of diastolic dysfunction and/or its progression towards HFpEF. Hence, this review will also describe the (potential) druggable therapeutic targets within these pathways, and where applicable, clinical trials intervening with these pathways.

## Clinical Associations Between Chronic Kidney Disease, Coronary Microvascular Dysfunction, and Heart Failure With Preserved Ejection Fraction

CKD is defined as a progressive decline of renal function and is associated with hypertension, proteinuria, and the loss of nephron mass (Noone and Licht, 2014). CKD is an independent risk factor for the development of HF, with increasing cardiovascular risk and mortality as renal function declines (Tonelli et al., 2006; Ronco et al., 2008). Additionally, HF is the major cause of death among patients with CKD (Kottgen et al., 2007; Bansal et al., 2017). Although renal dysfunction is present in about half of the patients with HF in general (Hillege et al., 2006; Smith et al., 2013), and is an important prognostic marker for adverse outcomes (Yancy et al., 2006; Ahmed et al., 2007; McAlister et al., 2012), particularly the association between HFpEF and CKD is very strong. In a cohort comparing patients with heart failure with reduced ejection fraction (HFrEF), HF with mid-range ejection fraction and HFpEF, renal dysfunction was associated with increased mortality in all HF subtypes, but was most prevalent in HFpEF (Streng et al., 2018). Gori et al. showed that 62% of the patients with HFpEF display abnormalities in at least one marker of renal insufficiency, with different markers correlating with different HFpEF phenotypes (Gori et al., 2014b). Further evidence for a causal relationship between CKD and HFpEF comes from a rat model, in which CKD was mimicked by nephrectomy of one whole kidney and two-third of the remaining kidney. Loss of nephron mass in these rats resulted in a cardiac HFpEF-like phenotype, with LV hypertrophy and diastolic dysfunction, but critical HFpEF features such as lung congestion and exercise intolerance were not reported (Sarkozy et al., 2019). In accordance with CKD as a causative factor for HFpEF, the majority of patients on hemodialysis display diastolic dysfunction and left ventricular hypertrophy, whereas overt systolic dysfunction and HFrEF are visible in only a minority of these patients (Hickson et al., 2016; Antlanger et al., 2017). In a prospective cohort study, 74% of the patients admitted for dialysis displayed left ventricular hypertrophy. In contrast, systolic dysfunction and left ventricular dilatation were present in only 15% and 32% of the patients, respectively (Foley et al., 2010). Left ventricular hypertrophy is not restricted to end stage CKD, but is already highly prevalent in the general CKD population (Collins, 2003). Indeed, the first visible myocardial alteration in patients with CKD is left ventricular hypertrophy (London, 2002), developing early in the progression of kidney dysfunction (Levin et al., 1996; Pecoits-Filho et al., 2012) and often co-occurring with myocardial fibrosis and diastolic dysfunction (Silberberg et al., 1989). Hypertension is an important predictor for development of left ventricular hypertrophy and HFpEF in patients with CKD (Levin et al., 1996; Thomas et al., 2008), while blood pressure reduction is associated with a lower cardiovascular risk (Blood Pressure Lowering Treatment Trialists Collaboration et al., 2013).

It should be noted however, that in addition to decreased diastolic function, both hemodialysis and pre-dialysis CKD patients show impaired regional systolic function measured by longitudinal, circumferential, and radial strain while ejection fraction was preserved (Yan et al., 2011). Similarly, patients with HFpEF can also display signs of systolic dysfunction defined by decreased global longitudinal strain and S′ velocity measured with tissue Doppler. Unger et al. showed in a large group of HFpEF patients that not only diastolic dysfunction, but also the severity of systolic dysfunction and mortality increased in parallel with CKD stage (Unger et al., 2016).

## Vascular Consequences of Chronic Kidney Disease

Arterial remodeling in CKD patients is characterized by arterial stiffening, increasing pulse pressure, as a consequence of premature aging, and atherosclerosis of the arteries (Laurent et al., 2006; Briet et al., 2012). Premature vascular aging is common in both CKD and HFpEF. Increased aortic stiffness has been strongly associated with both left ventricular dysfunction, and markers of renal dysfunction (Bortolotto et al., 1999; Borlaug and Kass, 2011), which precede and increase cardiovascular risk in patients with CKD (Kendrick et al., 2010; Middleton and Pun, 2010). Stiffer arteries result in an increased pulse pressure, as well as an increased pulse wave velocity, which cause the increased pulsatility to be transmitted into the microvasculature (Mitchell, 2008). Renal and coronary microvascular networks are very vulnerable to pulsatile pressure and flow, thus failure in decreasing pulsatility can result in damage of the capillary networks (Mitchell, 2008; Safar et al., 2015), and thereby contribute to coronary microvascular dysfunction. Fukushima et al. showed an impaired global myocardial flow reserve in CKD patients, even with a normal regional perfusion and function of the LV (Fukushima et al., 2012). Furthermore, coronary microvascular dysfunction was shown to be present in patients with end stage CKD (Bozbas et al., 2009), and was associated with an increased risk of cardiac death in patients with renal failure (Murthy et al., 2012).

Hypertension in CKD is thought to be mainly a consequence of volume overload due to increased sodium reabsorption by the kidneys (Charra and Chazot, 2003; Judd and Calhoun, 2015). Increased sodium loading might also contribute to HFpEF development independent of hypertension, through inducing a systemic pro-inflammatory state, which is detrimental to the coronary microvasculature (Yilmaz et al., 2012). Indeed, empagliflozin, a sodium glucose co-transporter-2 (SGLT2) inhibitor, initially developed as an anti-diabetic drug, resulted in decreased cardiovascular mortality in an initial type 2 diabetes cohort (Zinman et al., 2015). Interestingly, these effects seem to, at least for some part, be specific for empagliflozin as canagliflozin protected less against cardiovascular death (Neal et al., 2017). Although the mechanisms of action have not completely been elucidated yet, multiple pre-clinical studies are being conducted to investigate the myocardial effects of SGLT2-inhibitors (Uthman et al., 2018a,b). Currently, three mechanisms have been proposed to contribute to reduced cardiovascular mortality in patients receiving SGLT2-inhibitors in general and/or empagliflozin in particular (Bertero et al., 2018); (1) osmotic diuresis and natriuresis lower blood pressure and subsequently reduce left ventricular afterload; (2) empagliflozin may instigate a shift to cardiac ketone body oxidation, increasing mitochondrial respiratory efficiency and reducing ROS production; (3) empagliflozin can lower intracellular Na^+^ by inhibition of the cardiac Na^+^/H^+^ exchanger (NHE) and induce coronary vasodilation (Uthman et al., 2018a). The latter effect is especially promising as increased intracellular Na^+^, as present in failing cardiomyocytes, results in altered mitochondrial Ca^2+^ handling and subsequent ROS production, which may be ameliorated by SGLT2-inhibitors (Bertero et al., 2018). SGLT2-inhibitors, therefore, seem promising in the cardiorenal field as they are both cardio- and reno-protective (Butler et al., 2017). The effect of empagliflozin on cardiovascular mortality in HFpEF specifically, regardless of diabetic status, is being investigated in the ongoing EMPEROR-Preserved trial (ClinicalTrials.gov NCT03057951).

## Neurohumoral Consequences of Chronic Kidney Disease

CKD is associated with hyperactivation of the renin-angiotensin-aldosterone system (RAAS) in response to renal hypoxia resulting in volume overload (Nangaku and Fujita, 2008), which may contribute to the development and/or progression of HFpEF. Interestingly, testosterone can increase, whereas estrogen can lower renin concentrations (Fischer et al., 2002). Such protective effects of estrogen would especially be relevant in pre-menopausal women, and be lost in the typically older, post-menopausal female HFpEF population. Consistent with a detrimental effect of RAAS activation on HFpEF progression, RAAS activation can increase myocardial workload, by elevating systemic vascular resistance and left ventricular afterload, through vasoconstriction of systemic blood vessels in response to angiotensin II or by causing volume expansion due to increased sodium and water reabsorption in response to increased aldosterone levels (Brown, 2013; Forrester et al., 2018). It is not clear if angiotensin II can also induce myocardial cell hypertrophy and fibrosis independently of hypertension. Although *in vitro* studies have shown that there is a hypertension-independent effect of angiotensin-II on cardiomyocytes, multiple *in vivo* studies could not confirm these findings, suggesting that the effect of angiotensin II is blood pressure-dependent (Reudelhuber et al., 2007; Qi et al., 2011). Furthermore, RAAS-activation induces coronary microvascular endothelial dysfunction, through NADP(H)-oxidase activation and subsequent ROS formation (Bongartz et al., 2005; Wong et al., 2013). Myocardial perfusion might also be impaired by the vasoconstrictor effects of angiotensin II. During prolonged exercise, vasoconstriction occurs within metabolically less active tissues, mediated by angiotensin II and endothelin-1. Such response is inhibited in metabolically active tissues by NO and prostanoids, resulting in an efficient distribution of blood (Merkus et al., 2006). In a state of systemic inflammation, locally decreased NO bioavailability in the coronary microvasculature might result in disinhibition of angiotensin II-mediated vasoconstriction, resulting in reduced blood delivery to the heart.

Downstream from angiotensin II in the RAAS, aldosterone regulates blood pressure and sodium/potassium homeostasis through the mineralocorticoid receptor in the kidneys, by enhancing sodium reabsorption, thereby contributing to hypertension and high plasma sodium levels. Besides the renal effects, aldosterone has been shown to directly promote myocardial fibrosis, left ventricular hypertrophy, and coronary microvascular dysfunction, acting through endothelial and myocardial mineralocorticoid receptors, independently of angiotensin II (Brown, 2013).

RAAS inhibition is the preferred therapeutic strategy to slow down progression of renal failure and reduce proteinuria in CKD (Levin and Stevens, 2014). Despite the fact that most data show RAAS overactivation in HFpEF, clinical trials in HFpEF with drugs acting on the RAAS, have failed to improve (all-cause) mortality so far (Pitt et al., 2014; Zhang et al., 2016). It is, however, important to note that AT_1_-blockade with Irbesartan reduced mortality and improved outcome on cardiovascular endpoints in patients with natriuretic peptides below the median, but not in patients with higher natriuretic peptide levels (Anand et al., 2011), suggesting that RAAS inhibition may be beneficial in early HFpEF. Furthermore, *post hoc* analysis of the TOPCAT trial demonstrated geographically different effects of the mineralocorticoid receptor blocker spironolactone, with small clinical benefits in patients from America (Pfeffer et al., 2015). However, these patients were generally older, had a higher prevalence of atrial fibrillation and diabetes, were less likely to have experienced prior myocardial infarction, had a higher ejection fraction and had a worse renal function (Pfeffer et al., 2015), suggesting that a benefit of spironolactone was associated with a more HFpEF-like phenotype. A more recent *post hoc* analysis of this trial further showed that spironolactone did show an improvement in primary endpoints in patients with lower levels of natriuretic peptides and hence less advanced disease (Anand et al., 2017). Consistent with this suggestion, a recent meta-analysis showed that mineralocorticoid receptor antagonists do improve indices of diastolic function and cardiac structure in HFpEF patients (Kapelios et al., 2019). Interestingly, treatment of DM type 2 with mineralocorticoid receptor antagonists also improved coronary microvascular function (Garg et al., 2015). Altogether, these data suggest that intervening with the RAAS is beneficial in patients with less advanced HFpEF, whereas beneficial effects are lost in patients with more advanced disease. Therefore, clinical studies investigating HFpEF progression and clinical trials focusing on reducing or preventing progression of early HFpEF into advanced HFpEF need to be conducted.

Another approach intervening with the RAAS is the use of Entresto, an angiotensin receptor and a neprilysin inhibitor (ARNI), which is a combination of valsartan (AT_1_ receptor blocker) and sacubitril (neprilysin inhibitor). Neprilysin inhibition exerts its beneficial effects through inhibition of the breakdown of natriuretic peptides. Entresto was superior to the standard therapy, enalapril, in patients with HFrEF in reducing mortality and number of hospitalizations for HF (McMurray et al., 2014). In hypertensive rats with diabetes, ARNI reduced proteinuria, glomerulosclerosis, and heart weight more strongly than AT_1_ receptor blockade, and this occurred independently of blood pressure (Roksnoer et al., 2015, 2016). In a phase 2 double-blind randomized controlled trial in HFpEF patients, Entresto reduced NT-pro-BNP plasma levels and left atrial diameters to a greater extent than valsartan (Solomon et al., 2012). These findings led to the ongoing PARAGON-HF trial (ClinicalTrials.gov NCT01920711), which investigates the long-term effect (26 months) of Entresto compared to valsartan in HFpEF (Solomon et al., 2017).

Both CKD and HFpEF are accompanied by autonomic dysregulation (Salman, 2015). Sympathetic hyperactivity has a detrimental effect on both the heart and the kidney and aggravates hypertension and proteinuria. Furthermore, HFpEF patients show attenuated withdrawal of parasympathetic tone and excessive sympathoexcitation during exercise that cause β-adrenergic desensitization, chronotropic incompetence, and may thereby contribute to the limited exercise tolerance of these patients (Phan et al., 2010). A critical role for CKD in this process was suggested by Klein et al. (Klein et al., 2015), showing a clear correlation between CKD, decreased heart rate variability, chronotropic incompetence in HFpEF, and decreased peak VO_2_. Unfortunately, neither the SENIORS trial (van Veldhuisen et al., 2009), nor the OPTIMIZE-HF registry (Hernandez et al., 2009) showed a beneficial effect of beta-adrenoceptor blockade on all-cause mortality or cardiovascular hospitalizations. Furthermore, beta-adrenoceptor blockade failed to improve LV systolic or diastolic function in patients with ejection fraction >35%, as measured in the SENIORS echocardiography sub-study (Ghio et al., 2006). It should be noted that in the SENIORS trial ejection fraction cutoff was set at 35%, which is lower than current consensus about the cutoff of reduced and preserved ejection fraction. Additionally, in these studies, beta-adrenoceptor blockade was administered on top of existing medication, which often included RAAS-inhibitors. Conversely, in patients with treatment resistant hypertension, renal sympathetic denervation did improve diastolic function and reduce left ventricular hypertrophy, besides reducing blood pressure (Brandt et al., 2012), suggesting that there is indeed an interaction between CKD, sympathetic hyperactivity and diastolic cardiac function.

## Systemic Inflammatory Consequences of Chronic Kidney Disease

**Figure 2:**
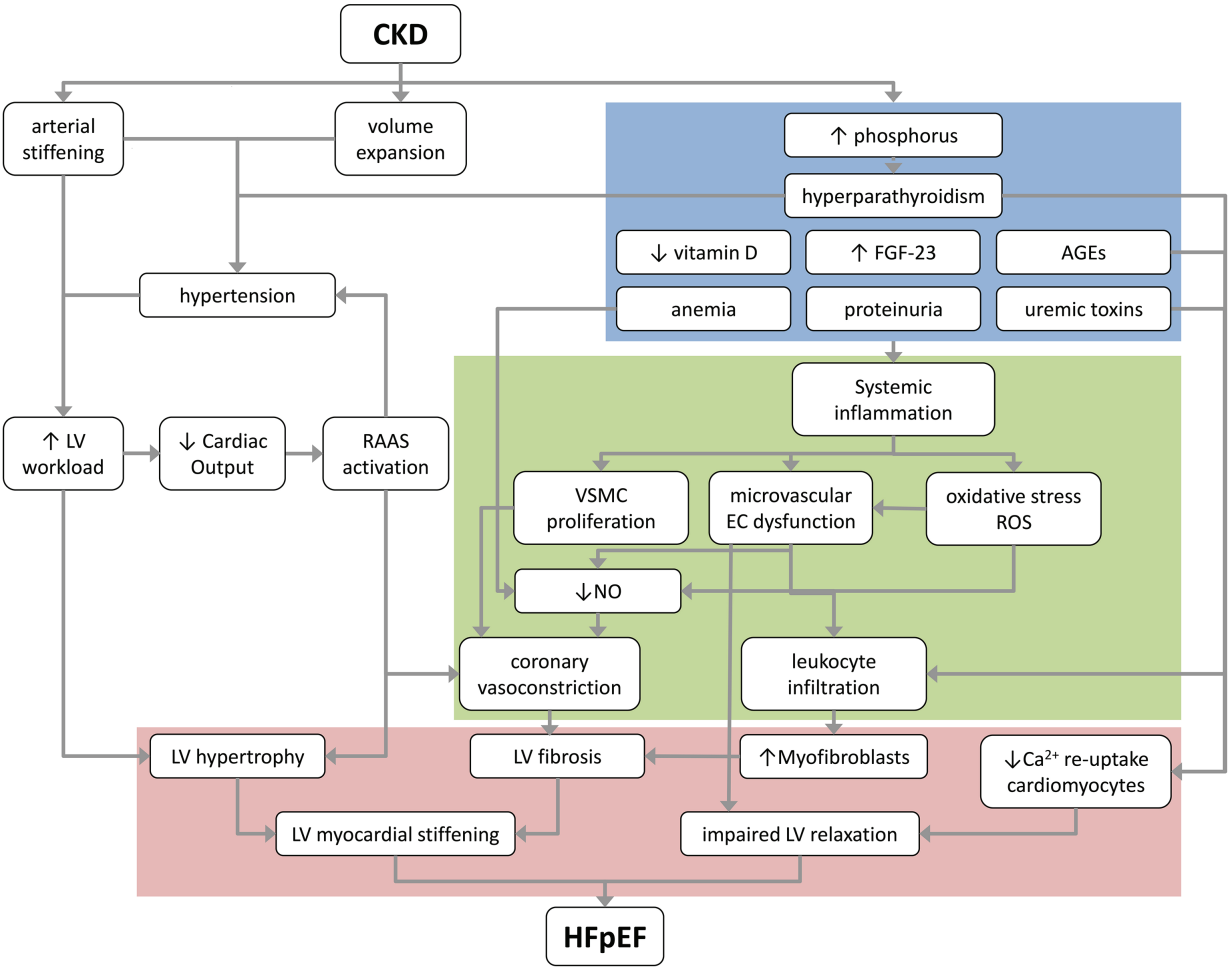
A proposed schematic overview of the pathological mechanisms that underlie the progression of CKD to HFpEF. Blue box depicts renal factors; green box depicts coronary microvascular factors; and red box depicts myocardial changes contributing to HFpEF. AGEs, advanced glycation products; CKD, chronic kidney disease; EC, endothelial cell; FGF-23, fibroblast growth factor 23; HFpEF, heart failure with preserved ejection fraction; LV, left ventricle; NO, nitric oxide; RAAS, renin-angiotensin-aldosterone system; ROS, reactive oxygen species; VSMC, vascular smooth muscle cell.

A pro-inflammatory state is already present in early stages of CKD (Stenvinkel et al., 2002), and is likely an important risk factor for cardiovascular morbidity and mortality on the long term (Ruggenenti et al., 2001; Sarnak et al., 2003). In HFpEF, a systemic pro-inflammatory state has been proposed to be a critical causal factor in coronary microvascular dysfunction as inflammatory cytokines can directly induce endothelial cell dysfunction, cause upregulation of adhesion molecules on coronary microvascular endothelial cells, and reduce NO bioavailability, resulting in impaired vasodilation and pro-fibrotic signaling (Figure 2; Rosner et al., 2012; Paulus and Tschope, 2013).

Targeting this pro-inflammatory state with 14 days of treatment with the recombinant human IL-1 receptor antagonist Anakinra, increased peak VO_2_, which correlated with a reduction in C-reactive protein (CRP) in the D-HART trial including 12 patients (Van Tassell et al., 2014). Unfortunately, prolonged treatment (12 weeks) in the follow-up D-HART2 trial in 28 patients did not increase VO_2_, despite small improvements in exercise duration and quality of life, as well as reductions in CRP and NT-pro-BNP compared to baseline values (Van Tassell et al., 2018).

It is possible that targeting systemic inflammation in general to ameliorate HFpEF is too broad to be successful. In the subsequent paragraphs, the contribution of the individual systemic factors: anemia, proteinuria, and reduced excretion of so-called uremic toxins as consequences of renal dysfunction and possible contributors to systemic inflammation, development of microvascular dysfunction, and HFpEF will be considered in more detail.

### Anemia

Anemia is an independent risk factor for development of HFpEF (Foley et al., 2010; Gori et al., 2014b), and is strongly associated with CKD (Thomas et al., 2008). Although hemoglobin levels decreased with worsening of kidney function in both patients with HFpEF and HFrEF, hemoglobin levels were slightly lower in patients with HFpEF as compared to HFrEF (Lofman et al., 2017). The main causes for anemia are iron deficiency and deficient erythropoietin (EPO) production in the renal tubular cells. In addition, urinary loss of red blood cells through enlarged fenestrations of endothelial cells in diseased glomeruli, hemolysis, vitamin B12 deficiency, hyperparathyroidism, and hemodilution may contribute to anemia in CKD patients (Westenbrink et al., 2007; van der Putten et al., 2008). Furthermore, the bone marrow erythropoietic response to EPO is impaired in CKD patients (van der Putten et al., 2008). Finally, the pro-inflammatory cytokine Il-6 can impair erythroid development, by inducing production of the iron regulatory peptide hepcidin by hepatocytes, increasing degradation of iron exporter ferroportin, and decreasing iron delivery to developing erythrocytes (Fraenkel, 2015). Hence, the systemic inflammatory state in CKD, but also in HFpEF, can aggravate anemia.

It is unknown whether anemia, iron deficiency, and/or reduced EPO are causal factors in the development of HFpEF or mere markers of CKD. The most obvious effect of anemia is a general reduction in O_2_ transport. In 75% of the HFpEF patients, peripheral oxygen consumption was impaired due to impaired diffusive oxygen transport and utilization (Dhakal et al., 2015). Hence, cardiac output needs to be increased to maintain systemic oxygen delivery. Both the consequent increase in myocardial work, and the reduced oxygen-carrying capacity of the blood may contribute to an impaired myocardial O_2_ balance. Such a disbalance between myocardial oxygen demand and supply is also present in ischemia with no obstructive coronary artery disease (INOCA), in which myocardial oxygen supply is limited by coronary microvascular dysfunction. Indeed, INOCA is increasingly being recognized as a risk factor for development of HFpEF (Crea et al., 2017; Obokata et al., 2018).

Anemia can also directly affect microvascular function as red blood cells can modulate microvascular tone (Cosby et al., 2003; Singel and Stamler, 2005). Red blood cells release NO, which is produced, particularly at low oxygen tensions, from deoxygenated hemoglobin and nitrite, to stimulate vasodilation, cGMP formation in smooth muscle cells and cardiomyocytes, and to inhibit mitochondrial respiration (Crawford et al., 2006). Thus, low levels of red blood cells simulate a condition of coronary microvascular dysfunction, with increased ROS and reduced NO, thereby inducing true coronary microvascular dysfunction and cardiomyocyte damage, which eventually can contribute to progression of HFpEF (Figure 2).

CKD patients on EPO therapy have shown signs of cardiovascular improvement and reversal of left ventricular hypertrophy (Goldberg et al., 1992; Frank et al., 2004), suggesting that correction of anemia may prevent progression of HFpEF. In addition to promoting red blood cell formation and correction of anemia, EPO can protect cardiomyocytes against ischemic injury and induce NO production by endothelial cells, thereby improving microvascular function (van der Putten et al., 2008). EPO can also have tissue protective properties by activating the EPO receptor and β common receptor, which are found in multiple peripheral tissues and are present on endothelial cells. EPO sensitivity can be increased by hypoxia but is decreased by a pro-inflammatory state, which is considered a hallmark of HFpEF; therefore, lower eNOS expression due to lower EPO or lower EPO receptors on the endothelium can contribute to the lower NO-bioavailability in the coronary microcirculation (Congote et al., 2010). Interestingly, in patients, EPO resistance is shown to be present in early CKD prior to the decrease in EPO levels that occurs in later stages of CKD (Mercadal et al., 2012). However, in a randomized controlled trial conducted in older adults with HFpEF, EPO supplementation with epoetin alfa did not improve left ventricular geometry or exercise capacity despite increases in hemoglobin levels (Maurer et al., 2013). One potential explanation would be that the 1.5 g/dl increase in hemoglobin in the treatment group was insufficient, particularly since the placebo-treated patients also showed a 0.8 g/dl increase in hemoglobin. Alternatively, decreased endothelial and/or cardiomyocyte sensitivity to, rather than too low levels of EPO and/or anemia are important in the progression of HFpEF (van der Putten et al., 2008). If so, it would be more beneficial to restore EPO sensitivity of specific cells rather than changing its levels. Reducing the pro-inflammatory phenotype of endothelial cells could potentially be beneficial in increasing endothelial EPO sensitivity. Alternatively, although not specific an enhancer of EPO sensitivity, targeting the protective tissue-specific effects of EPO might prove a viable therapeutic target, although to date, this was mostly evaluated in neurological disorders (Leist et al., 2004).

Iron deficiency, even without anemia, was also shown to be detrimental to the functional capacity of advanced HFpEF patients (Nunez et al., 2016), while diastolic dysfunction was not associated with functional iron deficiency (Kasner et al., 2013). Functional iron deficiency is detrimental to cardiomyocyte function as it reduces antioxidant capacity and limits oxidative phosphorylation thereby limiting energy production, potentially impairing energy-dependent Ca^2+^ reuptake during diastole (Anand and Gupta, 2018). Currently, iron supplementation with IV ferric carboxymaltose is being investigated in both anemic and non-anemic HFpEF patients in the FAIR-HFpEF trial (ClinicalTrial.org NCT03074591).

### Proteinuria

Proteinuria, an abnormal high protein concentration in urine, is present in up to 26% of CKD patients with an eGFR below 30 ml/min/1.73 m^2^ (Garg et al., 2002; Agrawal et al., 2009). Not only proteinuria, but also, more specifically, elevated urinary levels albumin, were associated with declining renal function (Klahr et al., 1994; GISEN Group, 1997; Brenner et al., 2001). Proteinuria is not just a marker of CKD, but also contributes to the exacerbation of CKD, by aggravating renal interstitial inflammatory cell influx resulting in interstitial fibrosis (Figure 2; Abbate et al., 2006; Ruggenenti et al., 2012).

In 1989, Deckert et al. already introduced the Steno hypothesis, which implies that albuminuria is not just reflecting local renal disease, but indicating more general endothelial microvascular dysfunction (Deckert et al., 1989). Indeed, large population based studies have shown that microalbuminuria correlates with a decrease in flow-mediated endothelium-dependent vasodilation in brachial arteries (Stehouwer et al., 2004), as well as in coronary arteries of diabetic patients (Cosson et al., 2006). In patients with essential hypertension, microalbuminuria was shown to correlate with levels of circulating von Willebrand factor, a marker for endothelial damage (Pedrinelli et al., 1994). Multiple studies have shown that (micro)albuminuria is highly prevalent in HFpEF, being associated with LV remodeling, and is a prognostic marker for further disease development (Miura et al., 2012; Brouwers et al., 2013; Katz et al., 2014; Gori et al., 2014b; Nayor et al., 2017). Consistent with a role for microalbuminuria as a prognostic marker for HFpEF, women with HFpEF are less likely to have albuminuria, while their eGFR is similar to that of men (Gori et al., 2014a), potentially explaining the better prognosis (±20% less likely to reach a MACE) in women with HFpEF (Lam et al., 2012). Furthermore, presence of CKD increased the risk for an all-cause event in women, to a similar risk present in men (Lam et al., 2012).

Currently, it is unclear, whether microalbuminuria simply reflects a more generalized microvascular endothelial dysfunction or may act as a causal contributing factor to HFpEF development by inducing coronary microvascular endothelial damage.

### Uremic Toxins

Insufficient glomerular filtration results in the retention of a variety of biologically active compounds in the blood, called uremic toxins. The accumulation of uremic toxins can have a deleterious effect on multiple organs, of which the cardiovascular system is most severely affected (Vanholder et al., 2008). Increased levels of uremic toxins are associated with an increased cardiovascular morbidity and mortality (Moradi et al., 2013). Moreover, blood urea nitrogen was shown to be an independent predictor for the progression from preclinical diastolic dysfunction to HFpEF, but not HFrEF (Zhang et al., 2017).

The mechanisms mediating the detrimental effects on the vascular system are multiple. The elevated uremia-associated pro-inflammatory cytokine levels, together with the associated chronic inflammatory state, can inhibit proliferation and enhance apoptosis of endothelial cells (Figure 2; Moradi et al., 2013). Furthermore, uremic toxins can increase von Willebrand factor levels, decrease NO bioavailability by inhibition of endothelial nitric oxide synthase (eNOS), and increase circulating endothelial microparticles (Brunet et al., 2011). Additionally, chronic low grade inflammation increases expression of adhesion molecules on endothelial cells and induces leukocyte activation with differentiation of fibroblasts to myofibroblasts, with subsequent production of collagen in the extracellular matrix, and migration and proliferation of vascular smooth muscle cells (Jourde-Chiche et al., 2011; Paulus and Tschope, 2013). Tryptophan-derived toxins can specifically activate the aryl hydrocarbon receptor pathway, and thereby induce endothelial dysfunction, and activate pro-fibrotic pathways in the myocardium, further enhancing inflammation and increasing vascular oxidative stress (Sallee et al., 2014). All these processes contribute to (coronary) microvascular dysfunction and remodeling.

Uremic toxins might also directly affect the left ventricular relaxation. Exposure of cardiomyocytes to uremic serum of CKD patients elicited inhibition of Na^+^/K^+^-ATPase, increased contractile force, impaired calcium re-uptake, and delayed relaxation (Figure 2; Periyasamy et al., 2001).

Elevated circulating and cellular levels of advanced glycation end products (AGEs) have been measured in patients with CKD (Stinghen et al., 2016). This is the result of impaired renal clearance of AGEs together with their increased formation resulting from oxidative stress and/or diabetes mellitus. Elevated circulating AGEs are linked to development and progression of both HFpEF and HFrEF (Hartog et al., 2006, 2007) and correlated positively with increased diastolic dysfunction in patients with diabetes mellitus type 1 (Berg et al., 1999).

In the LV, AGEs are particularly prominent in the coronary microvasculature, where their presence induces a pro-inflammatory phenotype (van Heerebeek et al., 2008), endothelial dysfunction by increasing oxidative stress and decreasing NO bioavailability and vascular stiffening by crosslinking of extracellular matrix (ECM) proteins (Smit and Lutgers, 2004; Hartog et al., 2007). In the myocardium, AGE-induced crosslinking of ECM proteins increases myocardial stiffness (Smit and Lutgers, 2004; Hartog et al., 2007). Furthermore, AGEs impair calcium handling in cardiomyocytes (Petrova et al., 2002). The latter is mediated by carbonylation of SERCA2a, which impairs its activity (Shao et al., 2011), as well as by enhancing calcium leakage from the sarcoplasmic reticulum through the ryanodine receptor (RyR2), thereby promoting mitochondrial damage and oxidative stress (Ruiz-Meana et al., 2019). Hence, reducing production and enhancing breakdown of AGEs could be a therapeutic option in HFpEF patients (Paulus and Dal Canto, 2018), particularly in patients with diabetes and CKD.

Besides glycemic control, there are three classes of drugs that can reduce AGEs: inhibitors of *de novo* AGE synthesis, drugs that break pre-existing AGE crosslinks and AGE receptor blockers (Zieman and Kass, 2004). Although, to our knowledge, none of these have been tested in HFpEF patients, treatment with aminoguanidine, a small hydrazine-like molecule capable of inhibiting AGE formation through interaction with and quenching of dicarbonyl compounds, resulted in a decrease of diabetes mellitus associated myocardial stiffening in rats, albeit without altering fibrosis (Norton et al., 1996). Furthermore, in DM type 2 patients, benfotiamine, a transketolase activator that blocks several hyperglycemia-induced pathways, prevented microvascular endothelial dysfunction and oxidative stress after an AGE rich meal (Stirban et al., 2006). Similarly, treatment with the AGE crosslink breaker alagebrium, improved endothelial function in patients with isolated systolic hypertension, which was associated with reduced vascular fibrosis and vascular inflammation (Zieman et al., 2007). For an overview of trials conducted with AGE-lowering therapies in CKD patients we refer to Stinghen et al. (2016). Some of these therapies which reduced AGEs in CKD patients might also be a viable chronic treatment option, to prevent or reverse AGE-associated microvascular dysfunction and subsequent diastolic dysfunction in HFpEF.

Lowering uremic toxin levels in general might also provide a viable, but challenging treatment option for HFpEF. The main challenges are to identify the specific uremic toxins that play a role in the pathogenesis of HFpEF, and to target a large variety of uremic toxins with just one class of drugs. Clinical trials with allopurinol, a therapy to decrease uric acid levels, resulted in slower disease progression and a decreased cardiovascular risk in patients with CKD (Goicoechea et al., 2010; Sezer et al., 2014). Even asymptomatic hyperuricemic patients may benefit from allopurinol treatment, as they showed improvements in endothelial function and eGFR (Kanbay et al., 2011).

## Consequences of Chronic Kidney Disease on Mineral Metabolism

### Vitamin D Deficiency

Declining renal function results in a reduced capacity to perform 1α-hydroxylation and in progressive loss of active vitamin D (Schroeder and Cunningham, 2000). Loss of active vitamin D subsequently leads to increased parathyroid hormone (PTH) production, so-called secondary hyperparathyroidism, eventually contributing to increased calcium, phosphate, and FGF-23 levels. In patients on hemodialysis, an association was reported between low vitamin D levels, systemic inflammation, and myocardial hypertrophy (Bucharles et al., 2011). Furthermore, low levels of vitamin D in these patients were related to increased cardiovascular mortality (Wolf et al., 2007; Drechsler et al., 2010; Bucharles et al., 2011). In non-dialysis CKD patients, lower vitamin D levels were shown to be associated with decreased flow mediated dilatation in the brachial artery, reflecting systemic endothelial dysfunction (Chitalia et al., 2012). Low vitamin D correlates with reduced coronary flow reserve in patients with atypical chest pain, suggesting that vitamin D also affects coronary microvascular function (Capitanio et al., 2013). Recently, in a large cohort of patients with diastolic dysfunction or HFpEF, lower vitamin D levels were associated with increased cardiovascular hospitalizations but not with 5-year mortality (Nolte et al., 2019). Furthermore, in a univariate analysis, calcidiol, but not its active metabolite, calcitriol, was associated with new onset HFpEF in the PREVEND study, but the association disappeared after adjustment for confounding variables (Meems et al., 2016). However, in patients with established HFpEF, vitamin D levels were lower as compared to healthy, sex-, race-, and age-matched controls, and inversely correlated with exercise capacity (Pandey et al., 2018).

In a trial of vitamin D supplementation by cholecalciferol therapy, reductions were observed in the left ventricular mass, inflammatory markers and brain natriuretic peptide levels of CKD patients on hemodialysis (Matias et al., 2010). In contrast, in the PRIMO-trial, 48 weeks of treatment with paricalcitol in a CKD cohort with preserved systolic function neither resulted in improved diastolic function, nor reduced left ventricular mass (Thadhani et al., 2012). However, cardiac MRI unveiled that just a minority of the included patients had left ventricular hypertrophy at baseline, possibly explaining the lack of a beneficial effect. Although the administration of vitamin D has positive effects through inhibition of PTH secretion, it also results in increased serum phosphate levels, with opposing effects (see next paragraph for details). When modulating vitamin D status, one should consider the use of vitamin D analogues, such as paricalcitol, which inhibit PTH synthesis, without substantially inducing hyperphosphatemia, providing promising therapies for restoration of vitamin D levels (Cozzolino et al., 2012).

### Phosphate and Parathyroid Hormone

In large cohorts of patients on hemodialysis, strong associations were found between serum phosphate, calcium, hyperparathyroidism, and an increased risk for overall cardiac mortality, elevated levels of cardiac injury markers, and a worse systolic and diastolic cardiac function (Block et al., 2004; Wang et al., 2014). Additionally, in a cohort of hospitalized patients with CKD, serum phosphate was related to elevated left ventricular concentric remodeling and diastolic dysfunction (Zou et al., 2016). Furthermore, in late stage CKD patients—on peritoneal dialysis—phosphate was independently associated with impairment of left ventricular diastolic function (Ye et al., 2016). At the structural level, elevated levels of phosphate (hyperphosphatemia) and PTH have been associated with the presence of hypertrophy and fibrosis of the LV specifically (Rostand and Drueke, 1999; Block et al., 2004). In addition, in a small cohort of patients on chronic hemodialysis, higher levels of calcium phosphate product were associated with higher CRP levels, and thus with a pro-inflammatory state. In this cohort, intensive lowering of phosphate levels resulted in lower CRP levels, and a significantly improved inflammatory status (Movilli et al., 2005).

Hyperphosphatemia can also directly induce coronary endothelial dysfunction (Di Marco et al., 2013), and also act directly on human vascular smooth muscle cells (VSMC), resulting in VSMC calcification (Jono et al., 2000). Furthermore, hyperphosphatemia can contribute to microvascular dysfunction and HFpEF pathogenesis by reducing prostaglandin synthesis (Ter Maaten et al., 2016). Prostaglandins synthesized in the blood vessel wall act as autocrine or paracrine factors and play a pivotal role in regulation of coronary microvascular function by exerting strong vasodilator effects and by inhibiting platelet aggregation. In clinical practice, supplementation of prostanoids is mostly used in patients with pulmonary hypertension. Prostacyclin analogues are available, such as Selexipag, an oral prostacyclin receptor agonist, which has vasodilator, antiproliferative, and antifibrotic effects. Currently, there is one trial ongoing, which investigates oral Treprostinil, a prostacyclin analogue, in pulmonary hypertension caused by HFpEF (ClinicalTrials.org NCT03037580).

PTH can cause left ventricular interstitial fibrosis and coronary microvascular dysfunction, *via* its inflammatory effects on monocytes and interstitial fibroblasts (Amann et al., 2003). Interestingly, primary hyperparathyroidism resulted in coronary microvascular dysfunction, which was restored after parathyroidectomy, underlining the effect of PTH on coronary microvascular function (Osto et al., 2012). In hemodialysis patients with secondary hyperparathyroidism, 20 weeks of treatment with cinacalcet ameliorated endothelial dysfunction, diastolic dysfunction, and cardiac hypertrophy by decreasing oxidative stress and increasing nitric oxide production (Figure 2; Choi et al., 2012).

### Fibroblast Growth Factor 23

Fibroblast growth factor-23 (FGF-23) is a hormone produced by osteoblasts and osteocytes, which inhibits phosphate reabsorption in the kidneys and suppresses circulating calcitriol, effectively lowering plasma phosphate levels in physiological conditions (Martin et al., 2012). In CKD, FGF-23 is no longer able to reduce phosphate levels due to loss of renal Klotho-FGF receptor 1 complex, resulting in both high phosphate and high FGF-23 levels (Komaba and Fukagawa, 2012). Elevated levels of FGF-23 are associated with an increased cardiovascular risk in patients with CKD (Negri, 2014), and with left ventricular hypertrophy in a cohort of CKD patients (Tanaka et al., 2016). These findings were confirmed in rats, where FGF-23 could directly induce left ventricular hypertrophy while ejection fraction was preserved (Faul et al., 2011). Furthermore, FGF-23 is associated with new-onset HFpEF in a large cohort study of people, who were free of cardiovascular disease at baseline (Almahmoud et al., 2018). Interestingly, in a cohort of HFpEF patients, FGF-23 was not associated with increased mortality, while this was the case for a cohort of HFrEF patients (Koller et al., 2015), suggesting that FGF-23 may be linked to disease onset rather than progression in HFpEF.

Mechanistically, FGF-23 induces chronic inflammation by stimulating cytokine secretion from the liver, but is also locally produced by M1 macrophages, and can thereby further modulate inflammation in the heart (Figure 2; Leifheit-Nestler and Haffner, 2018). FGF-23 inhibits ACE2, resulting in reduced degradation of angiotensin I and II into their vasodilator metabolites angiotensin-(1-9) and angiotensin-(1-7), (Leifheit-Nestler and Haffner, 2018) and consequently increased stimulation of AT_1_ receptors by angiotensin II. High levels of FGF-23 were further shown to cause endothelial dysfunction, increase superoxide formation, and decrease NO bioavailability in mouse aortas (Silswal et al., 2014). Finally, FGF-23 causes inhibition of 1α-hydroxylase, and can thereby contribute to microvascular damage and cardiac dysfunction due to vitamin D deficiency (Leifheit-Nestler and Haffner, 2018). Hence, elevated FGF-23 levels can contribute to development of HFpEF by attenuating coronary microvascular function and by enhancing angiotensin II induced vascular and myocardial fibrosis. Indeed, preliminary data of Roy et al., suggest that FGF-23 levels correlated with interstitial fibrosis in HFpEF (Roy et al., 2018). Furthermore, FGF-23 counteracted the beneficial effect of paricalcitol on left ventricular hypertrophy, by modulation of the calcineurin/nuclear factor of activated T cell (NFAT) pathway in a rat model of CKD (Czaya et al., 2019). FGF-23 inhibition with KRN23, an anti-FGF antibody, in open label phase 1/2 studies for X-linked hypophosphatemia, showed an increase in serum inorganic phosphate and active vitamin D in all subjects (Imel et al., 2015). Further research into a potential causal role of FGF-23 in HFpEF development is required, prior to embarking on therapeutic interventions.

## Conclusion

The kidneys and heart are interdependent organs that are highly connected through multiple systems on both macrovascular and microvascular level. Unfortunately, many studies on the cardiorenal connection have not been conducted in specific HFpEF populations. Pathological processes which are present in CKD, such as vascular changes, deficiencies in kidney produced factors, and impairments in renal filtration can cause and/or contribute to development of HFpEF *via* several processes, as summarized in Figure 2. Elevated levels of phosphate, PTH, FGF-23, AGEs and uremic toxins, but also anemia and proteinuria can induce a systemic pro-inflammatory state. This state can lead to left ventricular stiffening and coronary microvascular dysfunction by initiating endothelial cell dysfunction, oxidative stress, and vascular smooth muscle cell proliferation. Arterial stiffening, volume expansion, hypertension and RAAS activation, as consequences of CKD, increase left ventricular workload and hypertrophy.

The complexity and multitude of connections between the heart and kidney make it unlikely that there is a single causal contributor for progression from CKD to HFpEF. In addition, although HFpEF is more prevalent in women, and the effect of sex on cardiovascular disease is increasingly recognized (Regitz-Zagrosek, 2006), the specific role of sex in HFpEF pathology still needs to be identified. Multiple large trials have been conducted with treatments for HFpEF, targeting different pathophysiological processes, but unfortunately failed to show clinical benefit. Therefore, current guidelines on treatment of HFpEF focus on lifestyle interventions and the management of comorbidities such as diabetes mellitus, hypertension, obesity and CKD. In addition, it has been proposed that different HFpEF phenotypes exist that should be targeted with different therapeutic strategies. Both male and female CKD patients are interesting and easily identifiable subgroups of HFpEF patients, warranting further investigation both in pathogenesis, as in clinical trials to further investigate cardiorenal connection in HFpEF specifically, and to identify the unique mechanistic pathways involved in various phases of the disease.

## Author Contributions

This review was designed by JW, MB, and DM. JW and MB have written the largest body of text. OS, JJ, MV, DD, AD, and DM have made a substantial, direct, and intellectual contribution to specific topics. All authors approved the work for publication.

### Conflict of Interest Statement

The authors declare that the research was conducted in the absence of any commercial or financial relationships that could be construed as a potential conflict of interest.

## Abbreviations

AGE: advanced glycation endproduct
BNP: brain natriuretic peptide
cGMP: cyclic guanosine monophophate
CKD: chronic kidney disease
CRP: C-reactive protein
DM: diabetes mellitus
eGFR: estimated glomerular filtration rate
EPO: erythropoietin
FGF-23: fibroblast growth factor 23
HF: heart failure
HFpEF: heart failure with preserved ejection fraction
HFrEF: heart failure with reduced ejection fraction
LV: left ventricle
NO: nitric oxide
NT-proBNP: N-terminal prohormone of brain natriuretic peptide
PKG: protein kinase G
PTH: parathyroid hormone
RAAS: renin-angiotensin-aldosterone system
ROS: reactive oxygen species
TGF-β: transforming growth factor β
